# Role of vacancies, light elements and rare-earth metals doping in CeO_2_

**DOI:** 10.1038/srep31345

**Published:** 2016-08-24

**Authors:** H. Shi, T. Hussain, R. Ahuja, T. W. Kang, W. Luo

**Affiliations:** 1 School of Physics, Beijing Institute of Technology, 100081, Beijing, P. R. China; 2 Centre for Theoretical and Computational Molecular Science, Australian Institute for Bioengineering and Nanotechnology, The University of Queensland, Brisbane, Qld 4072, Australia; 3 Department of Physics and Astronomy, Uppsala University, Box 516, 75120 Uppsala, Sweden; 4 Department of Materials and Engineering, Royal Institute of Technology (KTH), 10044 Stockholm, Sweden; 5 Quantum Functional Semiconductor Research Center (QSRC), Dongguk University, Seoul 100715, Republic of Korea

## Abstract

The magnetic properties and electronic structures of pure, doped and defective cerium oxide (CeO_2_) have been studied theoretically by means of *ab initio* calculations based on the density function theory (DFT) with the hybrid HF/DFT technique named PBE0. Carbon (C), nitrogen (N), phosphorus (P), sulphur (S), lanthanum (La) and praseodymium (Pr) doped in CeO_2_ and CeO_2_ containing oxygen vacancies (*O*_*v*_) were considered. Our spin-polarized calculations show that C, N, Pr dopants and *O*_*v*_ defects magnetize the non-magnetic CeO_2_ in different degree. The optical band gap related to photocatalysis for pure CeO_2_, corresponding to the ultraviolet region, is reduced obviously by C, N, S, Pr impurities and oxygen vacancies, shifting to the visible region and even further to the infrared range. Especially, N-, S- and Pr-doped CeO_2_ could be used to photocatalytic water splitting for hydrogen production. As the concentration of *O*_*v*_ increasing up to 5%, the CeO_2_ exhibits a half-metallic properties.

CeO_2_, as one of the most important rare-earth metal oxides, has been in focus of intensive research during recent years due to its wide range of industrial applications. CeO_2_ special feature of high reactivity mediated by redox couple of Ce^4+^/Ce^3+^ could be used in catalysis^1^,^2^. Its unique oxygen storage capacity^3^ could make CeO_2_ material for solid oxide fuel cells^4^. Another potential application of CeO_2_ -based materials is in spintronics devices with advanced silicon based microelectronic devices^5^, due to an excellent match of crystal structure and high dielectric constant (*ε* = 26) of CeO_2_ with those of silicon^6^.

CeO_2_ is also a potential material for ultraviolet (UV) filtration^7^. CeO_2_ with a band gap of 3.2 eV, good transparency in the visible range, and no known toxicity, seems to be a promising inorganic material for use as a UV filter in sunscreen cosmetic products. Some previous studies regarding Zn-, Mg- and Ca-doped CeO_2_ demonstrated that the impurities shift the material’s band gap because of their effects on electronic transitions^8^. Furthermore, CeO_2_, as a wide band-gap semiconducting material which absorbs light in the near UV and slightly in the visible region, is a prospective material used in photocatalytic reactions, such as decomposing water to produce hydrogen and oxygen^9^. One of the most effective methods is doping. Zr- and La-doped CeO_2_ have found their potential application in solar cell devices^10^, Mao *et al.* demonstrated that N-doped CeO_2_ shows a visible-light absorbance shift^11^.

As mentioned above, the oxygen storage capacity of CeO_2_ makes it an important material. By releasing and storing oxygen during fuel-rich and lean conditions, an optimal oxygen pressure for the catalytic removal of harmful exhaust gases can be maintained. This is achieved by partial reduction/oxidation of the CeO_2_ and is related to the chemistry of oxygen vacancies in the material^12^. However, the description of oxygen vacancies in insulating transition and rare earth metal oxides is a challenge for modern electronic structure calculations^13^. In this paper, we used first-principles method to investigate the magnetic properties and electronic structures for doped and defective CeO_2_. Carbon (C), nitrogen (N), phosphorus (P), sulphur (S), lanthanum (La) and praseodymium (Pr) doped in CeO_2_ and CeO_2_ containing oxygen vacancies (*O*_*v*_) were considered.

## Results

### Magnetic properties

**Figure 1:**
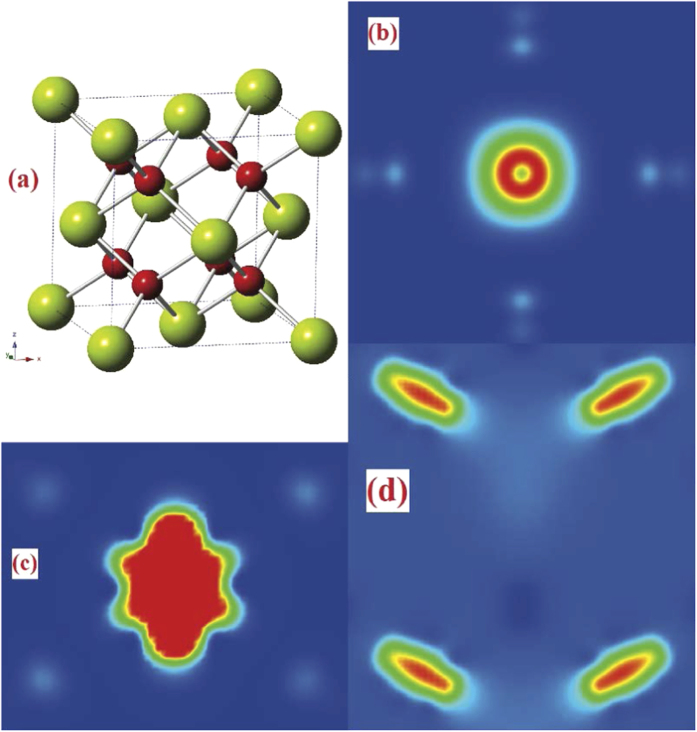
(**a**) CeO_2_ crystal with Fm
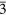
m structure (big green and small red spheres represent Ce and O atoms respectively). Spin-charge density (*n*_*α*_−*n*_*β*_) maps for C- (from the (001) side view) (**b**), Pr-doped (from the (110) side view) (**c**), and 1*O*_*v*_ (from (110) side view) (**d**) defective CeO_2_.

Cerium oxide (CeO_2_), or ceria, is a lanthanide oxide with a cubic fluorite structure (Fm
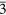
m) and a cell parameter of 5.41 Å at room temperature, in agreement with our PBE0 result of 5.49 Å, as shown in Fig. 1(a).

First, we invested the magnetic properties of pure, doped and defective CeO_2_. Table 1 lists the magnetic moment of doped and defective CeO_2_, and shows that C, N and Pr dopants and O vacancies (*O*_*v*_) magnetize the pure CeO_2_ in different degree. C impurity introduces a global magnetic moment of 2.0 *μ*_*B*_ per supercell. The C atom does the most contribution (0.733 *μ*_*B*_) to the total magnetic moment, and the four nearest Ce and six next-nearest neighboring O atoms provide 0.090 *μ*_*B*_ per Ce and 0.035 *μ*_*B*_ per O, respectively. Spin-polarized charge density calculation indicates that C 2*p* electrons diffuse to the second-nearest neighboring O atoms and lead to a polarization of surrounding O and Ce atoms, implying FM coupling between the doped C atom and its neighboring atoms (see Fig. 1(b)). N impurity magnetizes the pure CeO_2_ with a magnetic moment of 1.0 *μ*_*B*_ per surpercell, being smaller than that of C dopant. The N atom and six second-nearest O atoms contribute 0.563 *μ*_*B*_ and 0.048 *μ*_*B*_ per atom to the total magnetic moment respectively, whereas the spin-polarized contribution from the nearest Ce could be neglected. In a defective CeO_2_ system containing oxygen vacancies, two unpaired electrons localize in one *O*_*v*_, leaving cation dangling bonds. When the *O*_*v*_ site is occupied by the C or N dopant, the two unpaired localized electrons fulfill 2*p* orbitals of the dopant, and according to Hund’s rules, the substitutional C or N has four or five 2*p* electrons with high-spin configuration of 2*p*^4^ (↑↓↑↑) for C or 2*p*^5^ (↑↓↑↓↑) for N, which may create magnetic moment of 2.0 *μ*_*B*_ or 1.0 *μ*_*B*_ per impurity atom. Additionally, we found that P and S dopants do not produce spin-polarization in pure CeO_2_. For the P-doped case, according to effective charge calculation, 3*p* elections of the P impurity are much more delocalized than in the N-doped case, which leads to a vanishing of spin polarization.

We have also calculated La- and Pr-doped CeO_2_, to investigate the magnetic properties affected by lanthanide elements. From our calculations, La does not introduce spin polarization, whereas Pr magnetizes the doped CeO_2_ with a magnetic moment of 1.0 *μ*_*B*_ per supercell. For the Pr-doped CeO_2_, the localized 4*f* electrons do the most contribution (1.211 *μ*_*B*_) to the magnetization and the 2*p* electrons of the eight nearest O atoms occur opposite spin polarization with a non-negligible magnetic moment of −0.032 *μ*_*B*_ per O (see Fig. 1(c)). On the other hand, the La-doped CeO_2_ has no magnetizing phenomenon, and it is mainly due to the unpaired electron delocalization, being responsible for spin pairing.

Furthermore, we investigated the defective CeO_2_ system containing oxygen vacancies (*O*_*v*_). For the non magnetic CeO_2_, the formation of an *O*_*v*_ is known to result in the donation of two electrons which results in magnetism. Thus, upon *O*_*v*_ formation in CeO_2_, two electrons are left behind, and they localize on the *f*-level traps of the neighbouring Ce atoms, introducing two unpaired electrons which magnetize the defective CeO_2_. According to our calculations, the defective CeO_2_ containing 1, 2, 3 and 4 *O*_*v*_s introduces 2.0, 4.0, 6.0 and 8.0 *μ*_*B*_ magnetic moment per supercell, respectively. Our spin charge calculations demonstrate that the *f* electrons localizing on the neighbouring Ce atoms result in the spin polarization (see Fig. 1(d)).

### Electronic structures and photocatalysis

**Figure 2:**
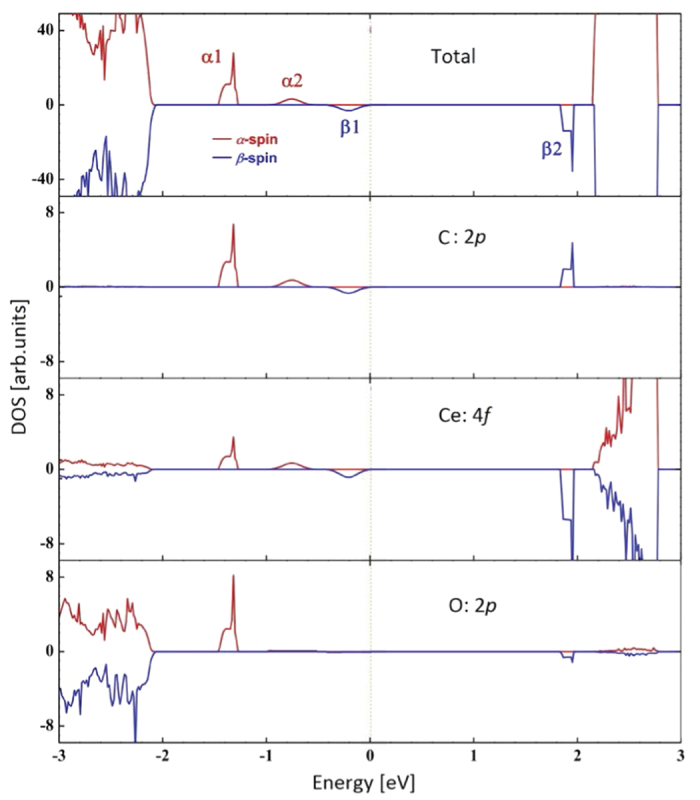
Total and partial spin polarized DOS for the C-doped CeO_2_. The Fermi level is set to zero.

Next, we mainly focus our discussion on band gaps and electronic structures of pure, doped and defective CeO_2_ systems. Our hybrid-DFT calculation for the band gap of pure CeO_2_ gives an overestimating value of 4.24 eV, compared to the experimental value of 3.2 eV^10^. Density of state (DOS) calculations demonstrate that the top of valance bands (VB) mainly consist of O 2*p* orbitals and the bottom of conduction bands (CB) primarily is formed by Ce 4*f* orbitals. Table 2 collects all the band gaps between the top of occupied states and the bottom of the unoccupied states for doped and defective CeO_2_. For the C-doped CeO_2_ system, four separated bands are introduced between the VB-CB gap. Because the C impurities magnetize CeO_2_, the band structure of C-doped system is polarized magnetically. Two bands are *α*-spin states and others *β*-spin states, as we can see in Fig. 2. Fermi level is located at the top of *β*1 band and the unoccupied *β*2 band is below the CB bottom, which leads to a reduced gap of 1.85 eV corresponding to an optical absorption. The two *α*-spin bands are all above the VB top and below the Fermi level, and the first possible optical absorption corresponds to an electron transition from the *α*2 band to CB bottom (2.68 eV) in *α*-spin state. So, the C impurities considerably reduce the forbidden gap of pure CeO_2_ by 37% for *α*-spin and 56% for *β*-spin states. When the band gap is reduced, electrons from the VB top can migrate easily to the CB bottom by absorbing light, forming electron-hole pairs. The electrons and holes that accumulate on the surface of material are then scavenged by oxygen molecules (O_2_) and hydroxides (OH^−^) dissolved in water to yield highly oxidative species, such as superoxide radical anions (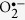) and hydroxyl radicals (^•^OH), which are responsible for decomposing and degenerating pollutants. Therefore, photocatalysis is closely related to the optical band gap of materials. C dopant shifts the optical response of CeO_2_ from the UV to the visible region and change the photocatalytic properties of CeO_2_.

CeO_2_ is not only a potential photocatalyst for water/air purification, but also a candidate used in photocatalytic water-splitting for hydrogen production. Both applications require essential photogeneration of electron-hole pairs, which is closely connected with the band gap. According to our calculations on C-doped CeO_2_, the bottom of unoccupied states of *α*- and *β*-spin are both higher (negative) than hydrogen production level (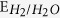) by 2.5 eV for *α*-spin and 2.2 eV for *β*-spin, meeting the requirement to initiate hydrogen production. On the other hand, the top of the occupied states located much higher than water oxidation level (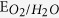) for both spin states, which does not satisfy the requirement for effective water oxidation. However, the *α*1 occupied band is close to the 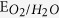, being slightly higher by 0.3 eV. Though it also does not meet the water oxidation requirement, quite small difference and the ineluctable errors from theoretical calculations make C-doped CeO_2_ a probable photocatalyst for hydrogen production.

DOS analysis shows that C and neighbour O 2*p* orbitals form the *α*1 band, and the unoccupied *β*2 band consists of C 2*p* and the neighbour Ce 4*f* orbitals. The C 2*p* states lead to the neighbour O 2*p* electrons and Ce 4*f* orbitals separate from the VB top and CB bottom respectively, forming hybridization orbitals, as we can see Fig. 2. Spin-polarized DOS calculations also can reveal the magnetization mechanism. The number of spin-up electrons should be more than that of spin-down states for a system with net magnetic moment. For C-doped CeO_2_, we found that there are obviously more *α*-spin electrons near the Fermi level, where the C 2*p*, neighbour O 2*p* and Ce 4*f α*-spin states are dominant. The integrated DOS calculations also demonstrated that the magnetism mainly results from the spin-exchange splitting between the *α*- and *β*-spin states near the Fermi level. Similar scenario is also observed in other C- or N-doped systems^14^.

**Figure 3:**
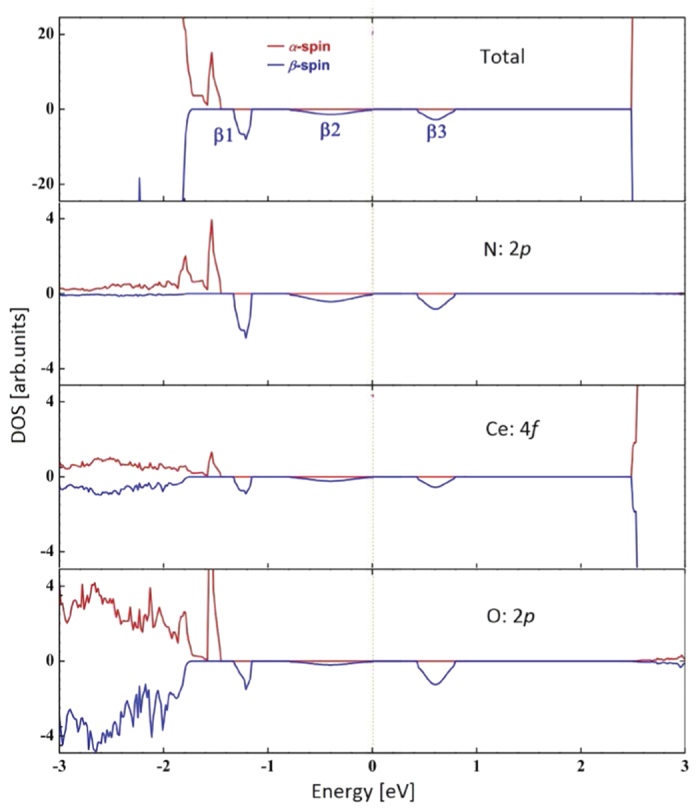
Total and partial spin polarized DOS for the N-doped CeO_2_. The Fermi level is set to zero.

Figure 3 shows that there are three separated *β*-spin bands introduced between the VB-CB gap and one *α*-spin band connected with the VB top for N-doped CeO_2_. Fermi level is at the top of *β*2 band. The gap related to the *α*-spin electron transition equals to 3.96 eV, being much larger than that of C-doped case and closing to the pure CeO_2_, whereas the corresponding gap for *β*-spin state is only 0.47 eV (see Table 2). Similar to the C-doped case, N 2*p* and the neighbour O 2*p* orbitals do the major contributions to the impurity bands. The N impurities pull the neighbour O 2*p* electrons from VB top towards Fermi level, however the affection on the neighbour Ce 4*f* orbitals could be neglected (see Fig. 3). Therefore, a degree of hybridization between the N 2*p* and neighbour O 2*p* states can be seen near the Fermi level. The *α*-spin gap related to photocatalysis meets the requirements for water spitting, whereas the slight reduction of the gap with respect to the pure CeO_2_ implies that photocatalytic water splitting occurs near the UV region. We found that occupied-*β*1 band approximately lies at the same level as 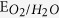, and unoccupied-*β*3 bands locates 0.46 eV above the 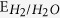. Therefore, the optical absorption corresponding to an electron transition from occupied-*β*1 to unoccupied-*β*3 bands (1.6 eV), which satisfies the conditions of photocatalytic water splitting, suggests that N-doped CeO_2_ could be applied in visible-region photocatalysis for hydrogen production.

**Figure 4:**
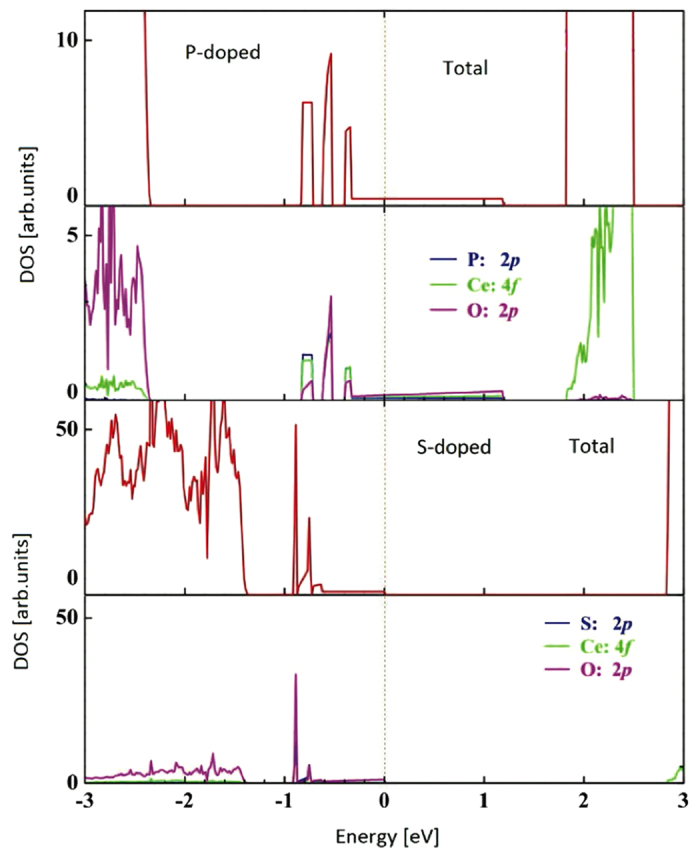
Total and partial DOS for the P- (up) and S-doped (down) CeO_2_. The Fermi level is set to zero.

P- and S-doped CeO_2_ do not exhibit the magnetization like C- and N-doped cases, thus the band structures are not spin-polarized. P impurities introduce three separated bands between the VB-CB gap. Two of them are narrow and below the Fermi level, and the third band is expanded and crosses the Fermi level with a tail, which shows a degree of metallic properties, as we can see in Fig. 4. For the S-doped CeO_2_ system, there are also three approximately separated bands introduced between the VB-CB gap. The two lower bands are narrow and the third impurity band is expanded but does not cross the Fermi level, unlike the P-doped case, still showing insulating properties (see Fig. 4). The gap between the top of occupied states and the bottom of unoccupied states is 2.85 eV, reducing the VB-CB gap of pure CeO_2_ considerably by around 33%. On the other hand, the first impurity band for the P-doped case locates above the VB top 1.55 eV, being much higher than that of the S-doped case (0.49 eV), which implies that P 3*p* orbitals are more delocalized. Similar to the C- and N-doped cases, for P- and S-doped CeO_2_, 3*p* orbitals of dopants, neighbour O 2*p* and Ce 4*f* orbitals hybridize to the impurity bands located between the VB-CB gap. For the S-doped case, our calculations show that the occupied impurity bands lie closely to the 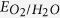 and the CB bottom is much higher than the 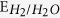 level, indicating a probability for water spitting.

**Figure 5:**
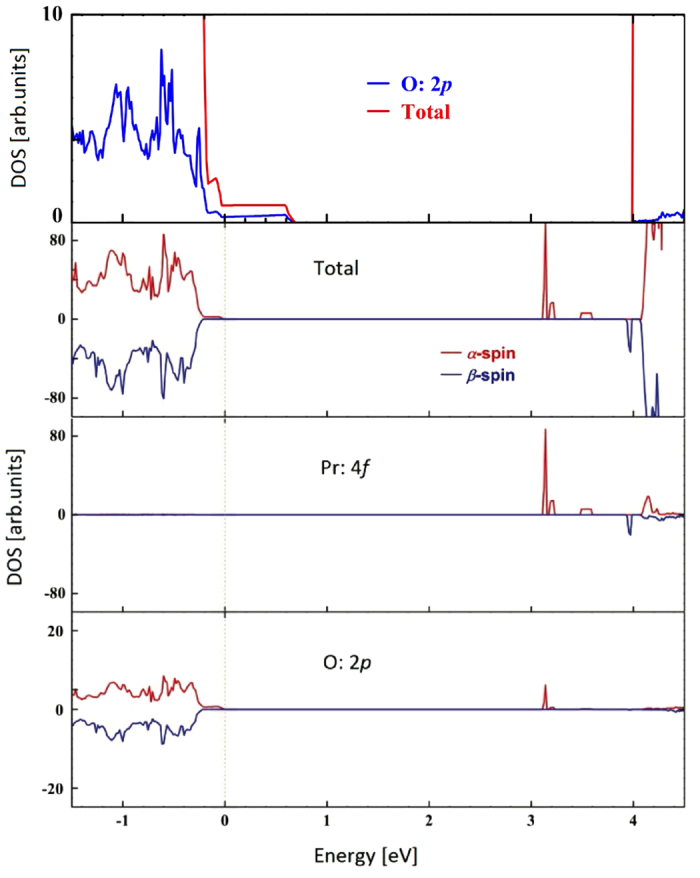
Total and partial DOS for the La- (up) and Pr-doped (down) CeO_2_. The Fermi level is set to zero.

Lanthanide impurities, such as La and Pr, change the band and electronic structures of pure CeO_2_. We found that unlike the above-mentioned dopants, La impurities do not introduce separated bands between the VB-CB gap. There are a few states crossing the Fermi level, exhibiting a little metallicity for the La-doped CeO_2_ system, as we can see Fig. 5. O 2*p* states forming the VB top in pure CeO_2_ are pulled towards the Fermi level by La 4*f* and 5*p* orbitals, leading to a degree of *p*-*f* hybridization straddling the Fermi level. The electronic structure of Pr-doped CeO_2_ is quite different from that of La-doped case. Pr dopants magnetize the doped CeO_2_ system and polarize the band structure. Four separated vacant bands are introduced between the VB-CB gap, being far from the Fermi level and closing to the CB bottom (see Fig. 5). Three of them are *α*-spin states and the other is *β*-spin. The three *α*-spin bands lie below the *β*-spin band. The gap between the top of occupied and the bottom of unoccupied states, corresponding to an optical absorption, is 3.12 eV for *α*-spin and 4.17 eV for *β*-spin states, reducing the VB-CB gap of pure CeO_2_ by around 26% and 2% respectively. Because of the obvious reduction of the optical gap in *α*-spin state, photocatalysis in the visible region is possible for the Pr-doped CeO_2_ case. Further, the top of occupied states locate 0.3 eV below the 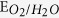 and 1.6 eV above the 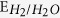 level, meeting the water-spitting requirements. Therefore, Pr-doped CeO_2_ also could be used in hydrogen production. DOS calculations demonstrate that these separated vacant bands mainly consist of Pr 4*f* orbitals and the contributions from other orbitals could be neglect, therefore the *f*-*p* hybridization is not remarkable for these bands. We also found that there are a few *α*-spin states moving out of the VB top towards the Fermi level (see Fig. 5). A slight *f*-*p* hybridization between Pr and neighbor O exists near the Fermi level.

**Figure 6:**
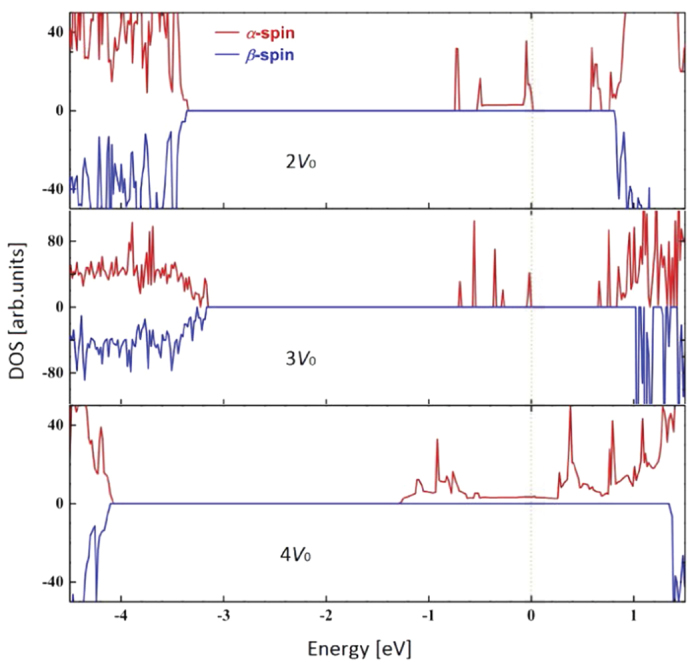
Total and partial spin polarized DOS for the CeO_2_ supercell containing 1*O*_*v*_. The Fermi level is set to zero.

Further, we performed calculations on the supercell containing 1, 2, 3 and 4 oxygen vacancies (labeled with 1*O*_*v*_, 2*O*_*v*_, …) to investigate the electronic structure of defective CeO_2_. For the 1*O*_*v*_ case, two connected bands with *α*-spin state lie 3.08 eV above the VB top, governing the Fermi level, and the gaps between the top of occupied and the bottom of unoccupied bands are 0.79 eV and 4.60 eV for *α*- and *β*-spin states respectively (see Fig. 6). So, the *O*_*v*_ reduces considerably the optical gap (4.24 eV) by around 81% in one spin state, whereas slightly increases it in the other spin state. Therefore, the photocatalysis of CeO_2_ containing oxygen vacancies may even shift up to the infrared region. The formation of an *O*_*v*_ is known to result in the donation of two electrons which form the defect bands locating between the VB-CB gap. According to our DOS calculations, the defect bands show mostly neighbour Ce 4*f* character. Then we can conclude that the two electron left behind localize on the *f*-level traps of the neighbour Ce atoms.

**Figure 7:**
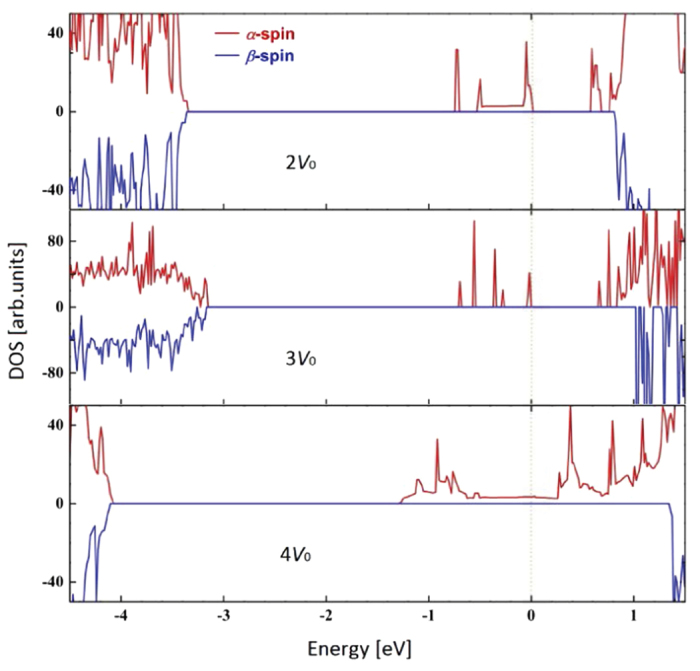
Total spin polarized DOS for the CeO_2_ supercell containing 2 (upper), 3 (middle) and 4 (lower) *O*_*v*_s. The Fermi level is set to zero.

For the 2*O*_*v*_ and 3*O*_*v*_ cases, there are several *α*-spin bands lying between the VB-CB gaps, as we can see Fig. 7. The gaps with *α*-spin state between the top of occupied and the bottom of unoccupied bands are 0.57 eV for the 2*O*_*v*_ case and 0.64 eV for the 3*O*_*v*_ case, even reducing the optical gap further. Both cases have separated vacant *α*-spin bands below the CB bottom. Finally, for the 4*O*_*v*_ case, corresponding to the defect concentration of around 5%, the defective CeO_2_ system shows an obvious half-metallic character (see Fig. 7). Therefore, the charge carriers within the defect bands are sufficiently mobile with an ideal 100% polarization, which meets the need for spin injection where a highly polarized spin current is desired.

## Conclusions

We have investigated magnetic properties, band and electronic structures of pure, doped and defective CeO_2_, such as C, N, P, S, La and Pr dopants, as well as *O*_*v*_ defects, in the framework of DFT using the hybrid functional technique PBE0. C, N, Pr impurities and *O*_*v*_ defects introduce magnetic moments of 2.0 *μ*_*B*_, 1.0 *μ*_*B*_, 1.0 *μ*_*B*_ and 2.0 *μ*_*B*_ per supercell with one dopant or defect, respectively. The calculations on the gap between top of occupied and bottom of unoccupied band, which corresponds to an optical absorption, demonstrated that C, N, S, Pr dopants and *O*_*v*_ defects reduces the band gaps evidently, showing a visible-light and even further infrared absorbance shift. It implies that the photocatalysis related closely to the optical absorption maybe occur in the visible region. Especially, N-, S- and Pr-doped CeO_2_ are promising photocatalysts for water splitting. As the concentration of *O*_*v*_ increasing up to around 5%, the CeO_2_ exhibits a half-metallic properties.

## Methods

We have studied the magnetic properties and electronic structures of doped and defective CeO_2_ by performing first-principles calculations based on the DFT. *Ab initio* simulations were performed using the projector augmented wave (PAW) method^15^ as implemented in the Vienna *ab initio* simulation package (VASP) code^16^,^17^. We have chosen the exchange-correlation functional proposed by Perdew *et al.* using the hybrid HF/DFT calculation denoted hereby PBE0^18^. For Ce atoms, we have used PAW potentials with following orbitals treated as valence states: 5*s*^2^5*p*^6^6*s*^2^4*f*^1^5*d*^1^ configuration. The calculations were performed using a cutoff energy of 500 eV and sampling the Brillouin zone with fixed Monkhorst-Pack *k* points (3 × 3 × 3) for conventional cell and (5 × 3 × 1) for a 72-atom (1 × 2 × 3) supercell.

## Additional Information

**How to cite this article**: Shi, H. *et al.* Role of vacancies, light elements and rare-earth metals doping in CeO_2_. *Sci. Rep.* **6**, 31345; doi: 10.1038/srep31345 (2016).

## Figures and Tables

**Table 1:** Magnetic moment (*μ*_*B*_) of doped and defective CeO_2_ per supercell.

Doped	C	N	P	S	La	Pr
Magnetic Moment	2.0	1.0	0.0	0.0	0.0	1.0
Defective	1*O*_*v*_	2*O*_*v*_	3*O*_*v*_	4*O*_*v*_		
Magnetic Moment	2.0	4.0	6.0	8.0		

**Table 2:** Band gaps (between the top of occupied states and the bottom of unoccupied states) of doped and defective CeO_2_ (*eV*).

Doped	C		N		P	S	La	Pr	
Spin	*α*	*β*	*α*	*β*				*α*	*β*
Gaps	2.68	1.85	3.96	0.47	—	2.85	—	3.12	4.17
Defective	1*O*_*v*_		2*O*_*v*_		3*O*_*v*_		4*O*_*v*_		
Spin	*α*	*β*	*α*	*β*	*α*	*β*	*α*	*β*	
Gaps	0.79	4.60	0.57	4.20	0.64	4.19	—	5.48	

For the spin-polarized cases, spin-up and spin-down states are labeled with *α* and *β* respectively. The supercell containing 1, 2, 3 and 4 oxygen vacancies are labeled with 1*O*_*v*_, 2*O*_*v*_, 3*O*_*v*_ and 4*O*_*v*_, respectively.
